# The optimal number of lymph nodes examined in stage II colorectal cancer and its impact of on outcomes

**DOI:** 10.1186/1471-2407-10-267

**Published:** 2010-06-08

**Authors:** Hok Kwok Choi, Wai Lun Law, Jensen TC Poon

**Affiliations:** 1 Department of Surgery, The University of Hong Kong, Queen Mary Hospital, Pokfulam Road, Hong Kong

## Abstract

**Background:**

Lymph node status is the most important prognostic factor for colorectal cancer. The number of lymph nodes that should be histologically examined has been controversial. The aims of this study were to assess the impact of the number of lymph nodes examined on survival of patients with stage II colorectal cancer and to determine the optimal number of lymph nodes that should be examined.

**Methods:**

The study included 664 patients who underwent resection for stage II colorectal cancer. The clinical and histopathologic data of the patients were prospectively collected and analyzed.

**Results:**

The median number of lymph nodes examined was 12 (range: 1 to 58). The 5-year disease free survival rate was significantly higher for patients with 12 or more lymph nodes examined compared to those with less than 12 lymph nodes examined. The significant difference in 5-year disease free survival persisted if the dividing number increased progressively from 12 to 23. However, the difference in survival was most significant (lowest *p *value and highest hazard ratio) for the number 21. The 5-year disease free survival of patients with 21 or more lymph nodes examined was 80% whereas that of patients with less than 21 lymph nodes examined was 60% (*p = *0.001, hazard ratio 2.08). Multivariate analysis showed that 21 or more lymph nodes examined was a factor that independently influenced survival. The 5-year disease free survival also increased progressively with the number of lymph node examined up to the number 21. After the number 21, the survival rate did not increase further. It was likely that 21 was the optimal number, at and above which the chance of lymph node metastasis was minimal.

**Conclusions:**

The number of lymph nodes examined in colorectal cancer specimen significantly influences survival. It is recommended that at least 21 lymph nodes should be examined for accurate diagnosis of stage II colorectal cancer.

## Background

Accurate assessment for the presence of lymph node metastasis is critical in predicting the clinical outcome of patients who have undergone radical surgery for colorectal cancer. The status of the lymph nodes also largely determines whether adjuvant chemotherapy should be given; such adjuvant chemotherapy has been shown unequivocally to provide disease-free as well as overall survival benefits in patients with node positive disease [1]. However, there is always a risk of disease understaging if the extent of lymph nodes assessment is sub-optimal. A tumor with lymph node involvement may be incorrectly classified as stage I or II if the number of lymph nodes examined is too small. The minimum number of lymph nodes that should be histologically examined for accurate staging of colorectal cancer has been controversial. While current guideline from the American Joint Committee on Cancer patient recommends the assessment of 12 lymph nodes or more [2], the recommendations in the literature range from 6 to 18 nodes [3-12]. The aims of this study were to assess the impact of the number of lymph nodes examined on survival of patients who underwent radical surgery for stage II colorectal cancer and to determine the optimal number of lymph nodes that should be examined.

## Methods

The study is a retrospective review and the protocol of 'Evaluation of clinical and pathological factors affecting outcomes following colorectal surgery' was approved by the Institution Review Board of the hospital. Between 2000 and 2006, a total of 664 patients underwent radical resection of colorectal cancer in the Department of Surgery, Queen Mary Hospital, The University of Hong Kong and their tumors were classified as stage II (T3-4N0M0) according to the 6^th ^edition of the TNM staging system of the International Union Against Cancer [13]. The clinical and histopathologic data of each patient were prospectively collected and entered into a computer database. The histopathologic features that were recorded included depth of tumor invasion (T3 or T4), tumor type (adenocarcinoma or mucinous carcinoma), tumor grade (well, moderately or poorly differentiated), lymphovascular permeation and number of lymph nodes examined.

Concerning examination of the surgical specimens, most of the specimens were fixed in 10% formalin and all were routinely processed for paraffin embedding. Lymph nodes in the specimen were identified by sight and palpation. Routine histopathologic examination was carried out using hematoxylin and eosin staining. No special fat clearance or staining techniques were routinely employed.

Adjuvant chemotherapy was given to patients with risk factors including emergency operation, mucinous carcinoma, lymphovascular permeation and less than 12 lymph nodes examined. All the patients were regularly followed up: every 3 months for the first 2 years, every 6 months for the following 3 years and then once a year. The median follow-up period was 44 months (range 12 - 104 months). Serum carcinoembryonic antigen level was measured regularly and imaging studies were performed in situations where recurrences were suspected.

The relationship between various clinical and histological variables and survival was evaluated using the Kaplan-Meier method. Differences between survival curves were tested for statistical significance by using log rank test. **The Cox proportional hazard regression model was used to identify the variables that could independently influence survival**. Concerning the number of lymph nodes examined, attempts were made to determine if there was a specific number at and above which the chance of lymph node involvement was minimal. Potential associations between the number of lymph nodes examined and other variables were also evaluated. Statistical Package for the Social Sciences, version 16.0 (SPSS Inc., Chicago, IL, USA) was used for all the statistical analysis. A *p *value of less than 0.05 was considered to be statistically significant.

## Results

**Figure 1:**
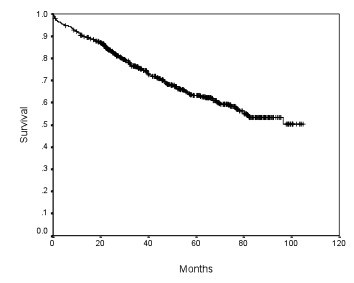
**Disease free survival of all the 664 patients**.

Of the 664 patients included, 385 were men and 279 women. Their age ranged from 27 to 96 years with a mean of 70 years. Location of tumors, urgency of operation (elective or emergency), depth of tumor invasion, tumor type, tumor grade and lymphovascular permeation were summarized in Table 1. The number of lymph nodes examined in each specimen ranged from 1 to 58 with a median of 12. The overall disease free survival of this group of patients was shown in Figure 1. The 5-year disease free survival rates in relationship to different clinical and histological variables were shown in Table 2. The 5-year disease free survival rate was significantly higher in patients underwent elective operation and in patients with T3 tumor. Mucinous carcinoma and tumors with lymphovascular permeation were associated with a significantly lower survival rate.

**Table 1:** Clinical and Histopathologic Data of All Patients

	Number of Patients
Location of tumors	
Ascending colon	131
Transverse colon	81
Descending colon	49
Sigmoid	161
Rectosigmoid junction	50
Rectum	192
	
Urgency of operation	
Elective	563
Emergency	101
	
Depth of tumor invasion	
T3	585
T4	79
	
Tumor type	
Adenocarcinoma	606
Mucinous carcinoma	58
	
	
Tumor grade	
Well differentiated	40
Moderately differentiated	580
Poorly differentiated	44
	
	
Lymphovascular permeation	
No	576
Yes	88

**Table 2:** 5-year Disease Free Survival Rates in Relationship to Different Clinical and Histopathologic Variables

	**5-year Disease Free Survival**	**p**
		
Sex		
Male	60%	0.103
Female	66%	
		
Tumor Location		
Proximal to splenic flexure	64%	0.364
Distal to splenic flexure	62%	
		
Urgency of operation		
Elective	65%	0.002
Emergency	50%	
		
Depth of tumor invasion		
T3	65%	0.000
T4	46%	
		
Tumor type		
Adenocarcinoma	70%	0.007
Mucinous carcinoma	61%	
		
Tumor grade		
Well or moderately differentiated	63%	0.438
Poorly differentiated	61%	
		
Lymphovascular permeation		
No	65%	0.000
Yes	45%	

**Figure 2:**
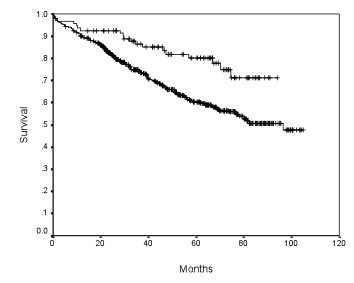
**Disease free survival of patients with at least 21 and less than 21 lymph nodes examined**.

The relationships between survival and different number of lymph nodes examined were evaluated. The 5-year disease free survival rate was significantly higher for patients with 12 or more lymph nodes examined compared to those with less than 12 lymph nodes examined. The significant difference in 5-year disease free survival persisted if the dividing number increased progressively from 12 to 23. However, the difference in survival was most significant (lowest *p *value and highest hazard ratio) for the number 21. (Table 3) The 5-year disease free survival rate of patients with 21 or more lymph nodes examined was 80% whereas that of patients with less than 21 lymph nodes examined was 60% (*p = *0.001, hazard ratio 2.08). (Figure 2) The 5-year disease free survival also increased progressively with the number of lymph node examined up to the number 21. After the number 21, the survival rate did not increase further. It was likely that 21 was the optimal number, at and above which the chance of lymph node metastasis was minimal. (Table 3)

**Table 3:** 5-year Disease Free Survival Rates in Relationship to Number of Lymph Nodes Examined

Number (N)	5-year disease free survival if lymph nodes examined < N	5-year disease free survival if lymph nodes examined ≥ N	P Value	Hazard ratio	95% confidence interval
8	56%	65%	0.281	1.18	0.89 - 1.55
9	57%	65%	0.322	1.14	0.86 - 1.49
10	56%	66%	0.055	1.29	0.99 - 1.69
11	58%	67%	0.074	1.26	0.97 - 1.64
12	57%	70%	0.015	1.39	1.06 - 1.82
13	57%	70%	0.006	1.46	1.11 - 1.93
14	57%	71%	0.005	1.49	1.12 - 1.98
15	59%	71%	0.011	1.46	1.08 - 1.97
16	59%	73%	0.004	1.61	1.67 - 2.22
17	59%	75%	0.003	1.72	1.21 - 2.44
18	59%	77%	0.003	1.93	1.31 - 2.84
19	59%	78%	0.002	1.99	1.32 - 2.99
20	59%	79%	0.002	2.05	1.33 - 3.16
21	60%	80%	0.001	2.08	1.36 - 3.33
22	60%	79%	0.006	1.92	1.19 - 3.12
23	61%	75%	0.045	1.64	1.00 - 2.69
24	61%	75%	0.058	1.71	0.99 - 2.93
25	61%	74%	0.085	1.65	0.92 - 2.95

The variables including urgency of operation, depth of tumor invasion, tumor type, lymphovascular permeation and 21 or more lymph nodes examined were put into Cox proportional hazard regression model. The results showed that all the variables except tumor type were independent factors that significantly influenced survival. (Table 4)

**Table 4:** Multivariate Analysis of Variables Showing Significant Association with Disease Free Survival

	Hazard Ratio	95% Confidence Interval	p
Urgency of operation	1.68	1.20 - 2.36	0.0025
Depth of tumor invasion	1.84	1.30 - 2.60	0.0006
Tumor type	1.98	0.96 - 3.96	0.0617
Lymphovascular permeation	2.09	1.48 - 2.96	0.0001
Lymph nodes examined ≥ 21	2.08	1.36 - 3.33	0.0025

The relationships between the number of lymph nodes examined and the other clinical and histological variables were shown in Table 5. Statistical analysis revealed that no specific variable had significant association with the number of lymph nodes examined. The number of lymph nodes examined in colon cancer specimen was also not statistically different from that of rectal cancer specimen (12.9 vs. 11.3 *p *= 0.1).

**Table 5:** Number of Lymph Nodes Examined in Relationship to Different Clinical and Histopathologic Variables

	Number of lymph nodes	P Value
Sex		
Male	11.84	0.168
Female	12.70	
		
Tumor Location		
Proximal to splenic flexure	12.89	0.654
Distal to splenic flexure	12.34	
		
Urgency of operation		
Elective	12.08	0.379
Emergency	12.84	
		
Depth of tumor invasion		
T3	12.36	0.136
T4	10.93	
		
Tumor type		
Adenocarcinoma	12.04	0.082
Mucinous carcinoma	13.98	
		
Tumor grade		
Well or moderately differentiated	12.12	0.395
Poorly differentiated	13.10	
		
Lymphovascular permeation		
No	12.21	0.907
Yes	12.10	

## Discussion

Lymph node involvement is the most important prognostic factor after radical surgery for colorectal cancer [14,15]. An accurate examination of the surgical specimen is mandatory to assess the lymph node status of the tumor correctly. Ideally all the lymph nodes should be harvested from the surgical specimen and examined in order to confirm that a tumor is node negative. At present, however, this goal is not practical. The actual number of lymph nodes that must be examined in the resected specimen has not yet been determined definitely. In the literature there is a lack of agreement in determining a universally valid minimum number of lymph nodes, above which there is no risk of understaging. Scott et al studied 50 cases of Dukes' C tumor and showed that 13 lymph nodes had to be examined to identify 94% of the specimen containing nodal metastases [3]. In 1990, the Working Party Report to the World Congress of Gastroenterology in Sydney recommended that a minimum of 12 lymph nodes should be examined [4]. Hernanz et al, based on a group of patients with colorectal cancer in which 75 were classified as Dukes' C, showed that if 6 lymph nodes were examined the probability to find at least a positive lymph node was 95 percent. The probability increased to 99 percent if 10 lymph nodes were examined. The authors concluded that 6 lymph nodes provided an accurate assessment of the presence of nodal metastasis and an optimal Dukes' B classification [5]. Goldstein et al and Maurel et al showed that the probability of correctly classifying a colorectal tumor as node positive increased with the number of examined lymph nodes. They also reported that this increase had a plateau. In their two series of patients, this plateau was reached when 17 lymph nodes in one and 16 in the other had been examined [6,7]. Tepper et al divided patients with stage II rectal cancer into four quartiles according to the number of lymph nodes examined and suggested that 14 lymph nodes needed to be studied to define nodal status accurately [8]. Cianchi et al found that the 5-year survival rate of stage II patients with eight or fewer lymph nodes examined was similar to that of stage III patients. Their results suggested that examining a minimum of nine lymph nodes per surgical specimen might be sufficient for reliable staging of lymph node negative tumors [9]. Swanson et al classified patients with T3N0 colon cancer into three groups according to the number of lymph nodes examined and found that a minimum of 13 lymph nodes should be examined to label the cancer as node negative [10]. Tsai et al reviewed a group of patients with T2-4N0M0 colorectal cancer and suggested that examining a minimum of 18 lymph nodes per surgical specimen might be taken into consideration for more reliable staging of lymph node negative cancer [11]. Vather et al analyzed various lymph node strata and showed a sharp and statistically significant drop in recurrence rate after the 16^th ^node mark. The recurrence rate remained at a low level for the remaining strata [12]. In our study, we demonstrated that the number of lymph nodes examined significantly influenced survival and the difference in survival was most significant when comparing patients with 21 or more lymph nodes and those with less than 21 lymph nodes examined. Indeed, 21 or more lymph nodes examined was an independent prognostic factor associated with better disease free survival. We also found that the 5-year disease free survival rate increased progressively with the number of lymph node examined up to the number 21. After the number 21, the survival rate did not increase further. It was likely that 21 was the optimal number, at and above which the chance of lymph node metastasis was minimal. We recommend that a minimum of 21 lymph nodes should be examined to label a tumor as node negative. The variability in searching ability for lymph nodes by pathologists and the different statistical methods employed are probably the major factors that explain the considerable variation among the different studies.

A proportion of patients in our study had adjuvant chemotherapy administered. Ideally this group of patients should be excluded when the survival rates were evaluated. However, in our practice adjuvant chemotherapy was only given to stage II patients with risk factors including emergency operation, mucinous carcinoma, lymphovascular permeation and less than 12 lymph nodes examined. These factors were all shown to be associated with lower survival rates. Therefore the inclusion of patients treated with chemotherapy should not affect the overall results.

The number of lymph nodes recovered from resection specimens is dependent on several factors. The variability in the number of lymph nodes in various regions of the large bowel and the extent of surgical lymphadenectomy alter the exact number of lymph nodes in a resection specimen. The diligence and skill of the pathologist in identifying and harvesting lymph nodes in the specimen determine the actual number of lymph nodes examined. It has been shown that nodal metastasis in colorectal cancer is often found in small lymph nodes (< 5 mm in diameter) [16,17], diligent search for lymph nodes is required on gross examination of resection specimens. Of note, many pathologists are uninformed about the necessity of examining a critical number of lymph nodes to accurately stage colorectal cancers. One Canadian study showed that only 58% of pathologists were aware of guidelines for lymph node retrieval in colorectal cancer at all and that as few as 25% knew that a minimum of 12 lymph nodes (National Cancer Institute guidelines 2000) is necessary for accurate designation of node negativity [18].

Lymph node collection from colorectal resection specimen is time consuming, particularly if the lymph nodes are small. Small lymph nodes are difficult to be found, especially amid large amount of mesenteric fat. Owing to the lack of widely accepted pathology practice standards for lymph node examination in colorectal cancer specimens, there are many variations in the basic pathologic techniques used for lymph node collection and submission for microscopic analysis. Some of these variations include the use of clearing solutions to improve visualization of small lymph nodes in the pericolonic or perirectal fat, the submission of one half versus both halves of each node for microscopic examination, and the preparation of one versus more than one tissue level per paraffin block of submitted nodal tissue. In an effort to reduce this variation, the College of American Pathologists has recommended that all grossly negative or equivocal lymph nodes be submitted in their entirety for microscopic examination. For grossly positive lymph nodes, it is recommended that a representative sample be submitted for microscopic confirmation. However, if fewer than 12 lymph nodes are found after careful gross examination, it is suggested that additional visual enhancement techniques that aid in the macroscopic identification of lymph node, such as fat clearing, be considered [3,19]. Unfortunately, the purchase and disposal costs for chemicals required for these techniques are high.

Hsu et al demonstrated that larger tumor, (tumor localization) and (depth of tumor invasion) were associated with a higher number (12 or more) of harvested lymph nodes in colorectal cancer specimen [20]. On the other hand, Horzic et al found that male gender, greater tumor size, better tumor differentiation as well as presence of acute inflammation in the abdominal cavity were independent predictors of increased number of examined lymph nodes [21]. According to the author, well-differentiated tumors were associated with increased number of examined lymph nodes. That might be due to better immune response against better-differentiated tumors. The association between acute inflammation in the abdominal cavity and number of lymph nodes examined might be explained by the fact that any inflammation causes reactive hyperplasia of lymph nodes. In our study, however, we could not identify any clinical or histological factor that influenced the number of lymph nodes examined.

Instead of exhaustive lymph node harvest and examination, an alternative way to determine lymph node status is sentinel lymph node biopsy. This approach has been extensively used for breast cancer and melanoma. However, the value of sentinel lymph node biopsy for colorectal cancer is still limited. A lot of studies have evaluated the role of sentinel lymph node biopsy in the management of colorectal cancer. At present sentinel lymph node biopsy has not been shown to be a reliable predictor of N0 status due to its relatively high false negative rate [22,23]. Formal lymphadenectomy and intensive lymph node examination are still essential for colorectal cancer.

## Conclusions

In summary, the number of lymph nodes examined in colorectal cancer specimen significantly influenced survival and the difference in survival was most significant when comparing patients with 21 or more lymph nodes and those with less than 21 lymph nodes examined. Twenty one or more lymph nodes examined was an independent prognostic factor associated with better disease free survival. The chance of lymph node metastasis was likely minimal if at least 21 lymph nodes showed no tumor involvement. We thus recommend that a minimum of 21 lymph nodes should be examined to label a tumor as node negative. The diligence and skill of pathologists remains an important factor that determines the actual number of lymph node examined. It is unlikely that sentinel lymph node biopsy can substitute for intensive lymph node examination in the management of colorectal cancer.

## Competing interests

The authors declare that they have no competing interests.

## Authors' contributions

WLL conceived of the study. HKC designed the study, performed the statistical analysis and drafted the manuscript. WLL revised the manuscript. All authors read and approved the final manuscript.

## Pre-publication history

The pre-publication history for this paper can be accessed here:

http://www.biomedcentral.com/1471-2407/10/267/prepub
